# ChIP-seq Analysis in R (CSAR): An R package for the statistical detection of protein-bound genomic regions

**DOI:** 10.1186/1746-4811-7-11

**Published:** 2011-05-09

**Authors:** Jose M Muiño, Kerstin Kaufmann, Roeland CHJ van Ham, Gerco C Angenent, Pawel Krajewski

**Affiliations:** 1 Applied Bioinformatics, Plant Research International, PO Box 619, 6700 AP Wageningen, The Netherlands; 2 Netherlands Bioinformatics Centre, PO Box 619, 6700AP Wageningen, The Netherlands; 3 Laboratory of Molecular Biology, Wageningen University, PO BOX 8128, 6700 ETPB Wageningen, The Netherlands; 4 Bioscience, Plant Research International, PO Box 619, 6700 AP Wageningen, The Netherlands; 5 Institute of Plant Genetics, Polish Academy of Sciences, 60-479 Poznań, Poland

## Abstract

**Background:**

*In vivo *detection of protein-bound genomic regions can be achieved by combining chromatin-immunoprecipitation with next-generation sequencing technology (ChIP-seq). The large amount of sequence data produced by this method needs to be analyzed in a statistically proper and computationally efficient manner. The generation of high copy numbers of DNA fragments as an artifact of the PCR step in ChIP-seq is an important source of bias of this methodology.

**Results:**

We present here an R package for the statistical analysis of ChIP-seq experiments. Taking the average size of DNA fragments subjected to sequencing into account, the software calculates single-nucleotide read-enrichment values. After normalization, sample and control are compared using a test based on the ratio test or the Poisson distribution. Test statistic thresholds to control the false discovery rate are obtained through random permutations. Computational efficiency is achieved by implementing the most time-consuming functions in C++ and integrating these in the R package. An analysis of simulated and experimental ChIP-seq data is presented to demonstrate the robustness of our method against PCR-artefacts and its adequate control of the error rate.

**Conclusions:**

The software *ChIP-seq Analysis in R *(CSAR) enables fast and accurate detection of protein-bound genomic regions through the analysis of ChIP-seq experiments. Compared to existing methods, we found that our package shows greater robustness against PCR-artefacts and better control of the error rate.

## Background

Genome-wide identification of *in vivo *protein-bound genomic regions is essential for a full understanding of transcriptional regulation. DNA fragments that are bound by proteins *in vivo *can be isolated by chromatin-immunoprecipitation (ChIP) and subsequently identified using microarrays (ChIP-chip) or high-throughput sequencing technologies (ChIP-seq). Recent studies [1,2] indicate that the ChIP-seq approach provides higher resolution and statistical power than ChIP-chip. To date, only two methods have been described for the analysis of ChIP-seq experiments in plants, i.e. [3] and the method developed by our group [2,4].

The common approach to analyze the millions of short sequence reads obtained in a typical ChIP-seq experiment is to map them to a reference genome using one of several mapping tools available, for example SOAPv2, Bowtie, or BWA [5-7]. Reads that map to multiple locations in the genome, so called 'multireads' [8], are often discarded to avoid the ambiguity of their genomic origin. To account for varying sequencing depths among the different samples in an experiment, current methods typically standardize the number of mapped reads across all samples by a scaling factor. However, it is becoming evident that more sophisticated normalization procedures are needed, since differences in coverage distribution among samples not only depend on the sequencing depth, but also on other properties of the sample [9], including methodological differences in library preparation, as well as biological differences in the chromatin state of the samples. We are aware of only two published ChIP-seq analysis methods that normalize the data to obtain the same coverage distribution across samples. The PeakSeq method [10] applies a scaling factor that is obtained from the linear regression between IP and control sample coverages, while in [11] a quantile normalization method is proposed. Here we describe the implementation of the approach introduced by our group [2], in which the statistical method of moments is used for the normalization process.

Subsequent to normalization, enrichment of genomic regions is commonly evaluated with a test statistic based on the Poissson or Binomial distribution. To control the false discovery rate (FDR) of such a test, it is necessary to obtain the distribution of the test statistics under the null hypothesis. Some methods, e.g. CisGenome [12], assume this distribution as known *a priori*, given the statistical properties of the test. However, this assumption strongly depends on how well the distribution used to construct the test statistics (e.g. Poisson distribution) can represent the real data. Another strategy is to try to empirically estimate the distribution of the test statistic under the null hypothesis; the most common method is to assume that the score values obtained in the comparison of the IP sample against the control will be a good estimation of the desired distribution. Examples of software that implement this approach are PICS [13], MACS [14] or QuEST [15]. A second problem with the use of the Poisson test is that the comparison of different ChIP-seq experiments is not straightforward, since the obtained scores or *p*-values will depend on the statistical power of each particular experiment (e.g. number of replicates, number of reads obtained, etc.). A review of existing algorithms is given in [8].

ChIP experiments typically yield low amounts of DNA and therefore require a high number of PCR amplification cycles prior to sequencing. This increases the probability of experimental artefacts, most importantly the uneven generation of high copy numbers of PCR fragments [16-18]. This effect in a given experiment can be estimated by measuring the percentage of non-unique sequence reads (hereafter referred to as "duplicate reads") obtained after sequencing. A high percentage of duplicate reads is an indication of potential problems due to PCR artefacts. Cell culture ChIP experiments yield larger amounts of DNA and can minimize the problem, but this approach can only rarely be used in plants. A typical Illumina-sequenced plant IP library usually yields around 30%-40% duplicate reads (Table 1, [2,3,19]), while cell culture samples in other organisms typically yield a low fraction of duplicate reads (5%-10% [20,21]). A possible approach to handle this problem is to identify and discard duplicate reads. However, in plant experiments, this can lead to a 30%-40% data reduction in a standard ChIP-seq experiment [2] (Table 1) and, consequently, to a decrease of the statistical power of the experiment. Also, it is expected that regions with a high read coverage will contain more duplicate reads than other regions of the same length, independently of PCR-artefacts. Therefore, the elimination of duplicate reads may incorrectly change the score ranking of these regions.

**Table 1:** Summary of read statistics for the ChIP-seq libraries analysed

Library name*	No. of sequenced reads	No. of mapped reads	No. of non-duplicated mapped reads	Percentage of duplicated mapped reads	SRA ID
S_c_	4,065,558	1,640,977	1,047,009	37%	SRX004992

S_1_	3,112,455	992,908	525,779	47%	SRX004990

S_2_-S_5_	NA	1,192,908	525,779	56%	NA

S_6_	614,236	124,619	56,619	55%	E-MTAB-587

S_7_	1,474,956	310,888	79,996	75%	E-MTAB-587

S_8_	4,105,326	1,558,098	78,434	95%	E-MTAB-587

A_c_	20,983,004	11,703,244	5,323,373	54%	SRX018394; SRX018395

A_1_	15,941,703	13,293,909	9,708,068	27%	SRX018392; SRX018393

* For library description see text

We present here an R package that implements the statistical methodology previously outlined by our group [2,4]. The method was developed to efficiently handle high-copy numbers of reads that result from PCR artefacts without the need of eliminating duplicated sequences. The coverage distribution of samples is normalized to obtain the same mean and variance across samples. Users can choose between Poisson or ratio-based testing. FDR control is achieved through the well-known method of permutations [22]. The most time-consuming functions are implemented in C++ and are fully integrated in the package. A comparison with three other publically available methods is presented in the context of plant ChIP-seq analysis.

## Implementation

The software accepts any plain text, tabular data format containing the following information for each mapped read: chromosome, location (bp), strand (+/-), read length (bp), and number of times mapped on the genome. Users can define specific input table formats in addition to the default option of the package, which expects the standard AlignedRead format supported by Bioconductor or the output of the mapping program SOAPv2. The average length of the DNA fragments subjected to sequencing must be provided by the user.

**Figure 1:**
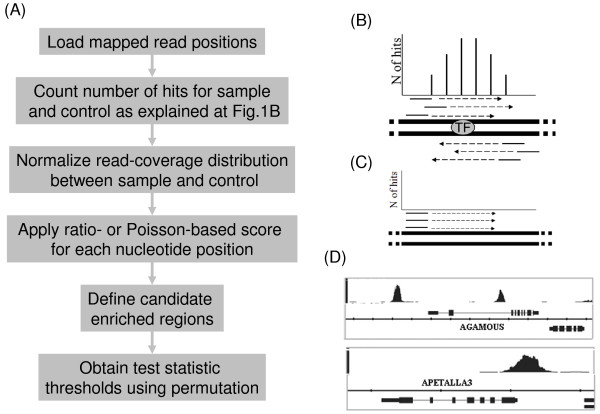
**CSAR analysis workflow**. (A) Typical analysis workflow using CSAR. (B) Mapped reads (continuous line) are virtually extended (dashed line) for each strand directionally. Number of extended reads that overlap each nucleotide position is counted for both strands independently, and the minimum value for both strands is taken as "number of hits". (C) Consequently, regions with duplicated reads mapping to only one strand will not be considered significant. (D) CSAR output can be visualized in a typical genome browser.

In an ideal ChIP-seq experiment, sequence reads that truly originate from a protein-bound genomic region should map in a 1:1 ratio to both strands of the chromosomal DNA (Figure 1B). However, because some sequences are represented by an artificially high number of duplicate reads due to PCR artefacts, this ratio can be distorted (Figure 1C). In the default setup of our package (Figure 1A), uniquely mapped reads are virtually extended to match the average length of the DNA fragments subjected to sequencing. The number of extended reads that overlap each nucleotide position *i *is then counted for both strands independently, and the minimum value for both strands is taken, providing counts *x*_*is*_, where *s *= 1,2 for control and IP sample, respectively (Figure 1B). Other setups allow the user to merge the information of both strands, or to just consider one of the strands in the analysis.

Prior to the estimation of read-enrichment in an IP sample relative to a control sample, the data need to be normalized to obtain equal read-coverage distributions. The two main factors affecting read coverage are:

1) Variable number of mapped reads among sequencing experiments. As commonly handled in the literature, the CSAR package allows normalization of the data by reporting the number of hits per *θ *millions of reads, where *θ *is an arbitrary number. Namely, the counts *x*_*is *_are transformed to

2) Variable number of regions sequenced. In the IP sample, reads will come preferentially from true positive and false positive protein-bound regions, while in the control sample, reads will come preferentially from false positive regions. This will result in different coverages in the IP and control samples that should be taken into account in the analysis [9]. In contrast to other packages, CSAR will normalize the read-coverage distribution in the IP sample to have the same mean and variance as the control sample. Namely, the observed *y*_*i2 *_are transformed to

where  and  denote the mean and standard deviation of *y*_*is*_.

After normalization, a score (*t*_*i*_) is calculated for each nucleotide position *i *on the basis of the Poisson-based or ratio (default) test.

For the Poisson-based test:

For the ratio test:

The parameter *β *represents the background coverage level of the IP sample after the value is scaled and normalized as any other value from the IP sample (see below). Usually, the coverage distribution of the control sample is not uniform with large regions showing no or very low coverage. These regions can be incorrectly declared significant since no good estimation of their coverage in the control can be obtained. To avoid this problem, the transformed counts in the regions with a coverage below *β *in the control sample are set to the value of *β*. The value of *β *is calculated as:

where *c *is a parameter representing the coverage level of the IP sample before scaling and normalization. The value of *c *can be given by the user, or calculated automatically (default option) as:

where *n*_*0 *_denotes the number of genomic positions for which *x*_*i2 *_> 0. In our experience, the ratio test gives more comparable results among different experiments, which is due to the fact that its score value is less dependent on the statistical power of the experiments as for the Poisson test.

Candidate peaks are defined as genomic regions with score values (*t*_*i*_) higher than a given cut-off. Candidate peaks separated by less than 100 bp (default parameter value) are merged. The maximum score value of the candidate peak is used as the test statistic value to test its significance.

In contrast to other packages, CSAR subsequently uses a permutation method to obtain the test-statistic threshold corresponding to a desired FDR level. Individual mapped reads are labeled as "control" or "IP" if they belong to either the control or IP sample, respectively. The labels are then randomly permuted between the mapped reads, and the new permutated datasets are subjected to the previously described ChIP-seq analysis. Since this permutation process removes any relationship between the mapped reads and the sample they came from [22], the score values obtained over a sufficient number of permutations will provide an accurate estimation of the score distribution under the null hypothesis that can be used to control the error rate, for example FDR.

CSAR can generate results regarding genomic positions of significantly read-enriched regions and their distance to annotated genomic features (e.g. genes, other annotated binding events) in tabular format. These can be directly used by other R functions or packages for further analysis or for graphical representation. The read-enriched genomic regions can be written to a UCSC web-browser compatible wiggle (wig) file and visualized (Figure 1D) with, for example, the Integrated Genome Browser [23]. The default parameters in CSAR are optimized for *Arabidopsis *ChIP-seq data, but they can easily be adjusted for other organisms.

## Results and Discussion

CSAR has been successfully used to analyze several plant ChIP-seq experiments and was shown to be computationally efficient and accurate [2,19]. Table 1 summarizes characteristics of Illumina sequence libraries that were reanalyzed in this study in order to compare the performance of CSAR (v1.4.0) with four other publicly available methods, i.e. QuEST (v2.4), PICS (v1.4.0), MACS (v1.4.0rc2) and Cisgenome (v1.2) [12-15]. SEPALLATA3 (SEP3) and APETALA1 (AP1) are two MADS-domain transcription factors involved in the regulation of floral development in *Arabidopsis thaliana*. Datasets S_1 _and S_c _represent an experimental IP and control libraries for a SEP3 ChIP-seq experiment [2]. S_6_, S_7 _and S_8 _represent sequencing libraries from the same IP experiment, except that low amounts of DNA were recovered from the ChIP step. Standard Illumina protocol was used for the library preparation. S_6_, and S_7 _were prepared according to the standard protocol and PCR amplified in 20 cycles. An additional second PCR amplification step (+10 cyles) was performed to the library S_8_. The amplification produced high numbers of duplicate reads (Table 1), with library S_8 _most affected. We used these libraries to evaluate the robustness of our method against PCR artefacts. Datasets S_2_-S_5 _represent *in silico *modifications of the S_1 _library. At random, 2000 uniquely mapped reads from S_1 _were amplified one hundred times each and added to the original S_1 _dataset. This process was repeated four times to generate the four dataset S_2_-S_5_. Datasets A_1 _and A_c _represent the IP and control libraries, respectively, combining two biological AP1 ChIP-seq replicates [19]. Libraries A_1 _and A_c _were sequenced on the Genome Analyzer II, the others on the Genome Analyzer I; all libraries were sequenced to a 36 bp read length. Table 1 summarizes the number of mapped reads, as well as the percentage of duplicate reads present in each dataset.

SOAPv2 (default parameters) was used to uniquely map reads to the *Arabidopsis *genome (ATH1.1con.01222004; ftp://ftp.arabidopsis.org/). Reads mapping to the chloroplast or mitochondrial genomes were discarded. Remaining reads were analyzed with default parameters at an FDR level <0.05 by CSAR, QuEST, PICS, MACS and Cisgenome [12-15] using the appropriate dataset as a control.

**Figure 2:**
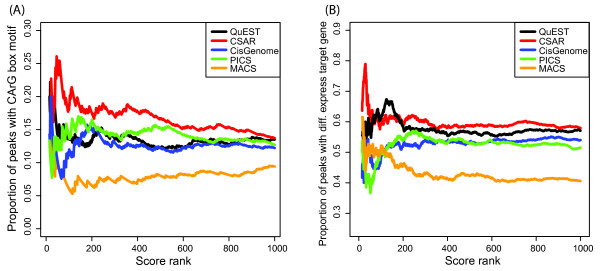
**ChIP-seq method comparison**. (A) Proportion of peaks with a CArG-box (CCW_6_GG or CCW_7_G) within a distance of 50 bp among the significant regions detected by each method in the comparison of S_1 _to S_c_. (B) Proportion of peaks detected by each method in the comparison of A_1 _to A_c _with at least one target gene differentially expressed. Only peaks near a gene (3 kb upstream or 1kb downstream) represented in the microarray experiments were considered. The list of genes which expression is affected by AP1 was downloaded from [19], we used the list denoted "Agilent and_or Operon_BH-0h". Default options for QuEST results in the identification of only 66 significant peaks, therefore we used the option "Relaxed peak calling parameters" for Figure 2B. For comparison purposes, all scores reported by the different methods were transformed into rank scores with zero as the rank of the most significant peak.

Figure 2A shows the proportion of significant SEP3 peaks declared by each method and for which a CArG box motif was present at a maximum distance of 50bp. Note that the CArG box is the known DNA binding motif of MADS-domain transcription factors and can thus be used as a validation criterion. CSAR shows a stronger enrichment than other methods.

For AP1, publically available gene expression data could be used to validate peak calling. The expression data was generated in AP1 induction experiments on the same tissue that was used in our AP1 ChIP-seq experiment [19]. Figure 2B shows the percentage of significant AP1 peaks declared by each method close to at least one potential direct target gene, where the target genes were as the ones which were differentially expressed in the time-series gene expression data [19]. CSAR shows a stronger enrichment than other methods.

In order to study the robustness of each method against PCR artefacts, we considered the regions declared as significant in the comparison S_1 _to S_c _for each evaluated method as its gold standard. A high percentage of regions declared significant in the analysis of the *in silico *(S_2_-S_5_) or experimentally (S_6_-S_8_) modified S_1 _libraries but not present in the gold standard for each method will indicate a lack of robustness. Table 2 gives the number of significant regions in the different datasets as detected by each method. The number of significant regions in common in the comparison of S_1 _to S_c _is shown, as well as the percentage of False Positives. A "common region" is defined as a significant region (FDR < 0.05) located within 250 bp of a significant region (FDR < 0.05) in the comparison of S_1 _to S_c_, using the same software; these common regions are considered as True Positives to allow for calculation of the percentage of False Positives. On average, 2,365 regions were declared as significant in the comparison of S_1 _to S_c _by the five methods. CSAR declares more regions significant than the other methods do.

**Table 2:** Number of significant regions detected

		S_1 _vs S_c_	S_2_-S_5 _vs S_c_*	S_6 _vs S_c_	S_7 _vs S_c_	S_8 _vs S_c_
CSAR	Total	3,235	3,306(5)	57	150	126
	
	Common	3,235	3,226(2.6)	52	130	104
	
	False Positives	-	2%	9%	13%	17%

QuEST	Total	985	989(11)	5,663	4,724	5,709
	
	Common	985	971(4.2)	440	433	422
	
	False Positives	-	2%	92%	91%	92%

CisGenome	Total	2,030	14,632(30)	9	91	169
	
	Common	2,030	1,633(4)	1	24	23
	
	False Positives	-	89%	89%	74%	86%

PICS	Total	2,846	1,952(24.7)	1,256	1,575	153
	
	Common	2,846	1,253(5.9)	382	435	51
	
	False Positives	-	36%	70%	72%	67%

MACS	Total	2,728	2,728(0)	2,821	3,687	3,624
	
	Common	2,728	2728(0)	631	761	716
	
	False Positives	-	0%	78%	79%	80%

*Results for the *in silico*-modified libraries (S_2_-S_5_) are summarized with its average and standard deviation (in parenthesis)

In the analysis of the *in silico *modified libraries S_2_-S_5_, MACS, CSAR and QuEST are the most robust methods with respect to the presence of high numbers of duplicate reads, as indicated by the low percentages of False Positives, an error rate below the 5% FDR control desired. A possible cause for the high percentage of False Positives obtained by Cisgenome in our *in silico *modified datasets might be in its FDR estimation step. Cisgenome assumes a Negative Binomial or a Poisson distribution for the score distribution under the null hypothesis. However, the presence of high numbers of duplicate reads will modify its original distribution and will have a strong effect on the FDR estimation.

In the case of the experimental libraries which had high levels of duplicate reads (S_6_, S_7 _and S_8_), CSAR clearly shows a lower percentage of False Positives than all other packages, with an error rate close to the desired 5% FDR control. Because one might argue that this is done at the cost of having a relatively small number of significant regions declared in comparison to other packages, we repeated computations in CSAR with a more relaxed error control that gave 80% of false positives (a rate similar to the one actually obtained for MACS). In this way, 717 true positive (common) regions were found for S_6 _(out of 3,597 significant regions), 771 for S_7 _(out of 3,737), and 655 for S_8 _(out of 3,307), which is comparable with the number of true positives obtained with MACS. It is interesting to note that although MACS shows 0% of False Positives in the *in silico *libraries, in the experimental libraries, the error increases to an average of 79%. MACS (default options) eliminates reads that map to the exact same positions and strand above a maximum number. For this reason, MACS eliminates the reads added *in silico *since these have the same sequence and therefore the same position and strand. In the experimental libraries, this strategy apparently did not work out. We hypothesize that due to degradation of the DNA fragments subjected to sequencing or due to sequencing errors, the short reads obtained from fragments with the same sequence will not always have the exact same positions, preventing MACS from eliminate them. In the CSAR approach this is not a problem because it requires both strands to support the binding event independently.

Since the percentage of duplicate reads can be easily calculated, we advise to always report it as a measure of quality in future ChIP-seq experiments. In this study we used the libraries S_6 _- S_8 _as extreme examples of the effect of PCR artefacts, but we advise in general against working with high levels of duplication in a normal ChIP-seq experiment. Further study should establish more precisely which levels of duplication are still acceptable. This should be done in combination with evaluating other parameters such as the number of mapped reads. When working with proteins that bind preferentially to promoter regions, we found it useful to graphically represent for each experimental library the distribution of distances (bp) between the position of read-enriched regions and the start position of genes; in such graphs one should typically see enrichment in the expected positions (e.g.: promoter regions for SEP3 and AP1 TFs). If this is not the case, this might be an indication of a problem in the experimental IP enrichment. CSAR provides functions to easily calculate and visualize this distribution and to report the number of duplicate reads.

In conclusion, the CSAR package, implemented in the popular R language, provides an accurate and efficient tool for the analysis of plant ChIP-seq data. It shows better accuracy compared to other methods in the two plant ChIP-seq experiments considered, and, in particular, it shows a high level of robustness against PCR-artefacts. A good error rate control is one of the most important features of any statistical process, and CSAR shows a good control even with a high percentage of duplicate reads.

## Availability and requirements

• **Project name**: CSAR

• **Project home page**: http://bioconductor.org/packages/release/bioc/html/CSAR.html

• **Operating system(s)**: Platform independent

• **Programming language**: R

• **Other requirements**: R version 2.8.1 or superior

• **License**: Artistic-2.0

• **Any restrictions to use by non-academics**: None

• The software (source code) and examples are attached in Additional file 1. It can also be downloaded via the project home page.

## Competing interests

The authors declare that they have no competing interests.

## Authors' contributions

JMM implemented the R code, evaluated the program and wrote the manuscript. KK generated the experimental data. JMM and PK developed the statistical method. All authors participated in the design of the study, and read and approved the final manuscript.

## Supplementary Material

Additional file 1 **CSAR R package source**. The R package source for CSAR (version 1.4.0) is included as additional file. Click here for file
