# Supplementary material for: ChIP-seq Analysis in R (CSAR): An R package for the statistical detection of protein-bound genomic regions
Source: Plant Methods. 2011 May 9;7:11. doi: 10.1186/1746-4811-7-11 (PMC3114017; doi:10.1186/1746-4811-7-11)
Supplement: Additional file 1 — CSAR R package source. The R package source for CSAR (version 1.4.0) is included as additional file. [file 1746-4811-7-11-S1.GZ › CSAR/inst/doc/CSAR.pdf]

An Introduction to the CSAR Package

Jose M Muino*

November, 2009

Applied Bioinformatics
Plant Research International, Wageningen UR
Wageningen, The Netherlands

1 Introduction

We present here a R package for the statistical detection of protein-bound genomic regions, where, considering the average DNA fragment size submitted to sequencing, single-nucleotide enrichment values are obtained. After normalization, sample and control are compared using a test based on the Poisson distribution. Test statistic thresholds to control false discovery rate are obtained through random permutation. The computational efficiency is achieved implanting the most time-consuming functions in C++ language, and integrating them in the R package. Standard outputs of the package are tables of genomic coordinates of significantly enriched region locations, level of enrichment per nucleotide position and the distance of enriched regions to annotated genomic features. The algorithm is described in detail in [1], [2], [3].

2 Methods

Due to PCR artifacts a high number of reads can represent the same sequence. The elimination of these duplicated reads usually leads to a 15%-25% data reduction in a standard plant ChIP-seq experiment. This artifact is strand dependent, therefore CSAR requests that the number of extended reads that overlap a given nucleotide position should be supported by both strands independently. This is achieved by virtually extending the mapped reads to a length of 300 bp (the average DNA fragment length submitted to the sequence process) for each strand independently, and after taken the minimum value for both strand at each nucleotide position.

Before test enrichment between sample and control, the number of overlapped reads distribution of the sample is normalize to have the same mean and

*jose.muino@wur.nl

variance that the control.. Subsequently, a score enrichment is calculated based on the Poisson test or in the ratio.

Permutation is applied to calculate the FDR thresholds. The mapped reads are randomly permutated between the control and sample group, and new scores are calculated for this new permutated dataset. The procedure is repeated until have a enough number of permutated scores. This scores are used to calculate the FDR thresholds.

3 Example

We will use a dataset included in the CSAR package for this demonstration. The data represent a small subset of a SEP3 ChIP-seq experiment in *Arabidopsis* [1].

First, we load the CSAR package and the data *CSAR-dataset*. We use the *mappedReads2Nhits* function to calculate the number of hits (number of extended reads that overlap a particular position) per nucleotide position for the control and sample dataset. The results for each chromosome are saved in a file with the name of the chromosome and the tag used in the parameter *file* See function *mappedReads2Nhits* for more information about the parameter values.

```
R> library(CSAR)
R> data("CSAR-dataset")
R> head(sampleSEP3_test)

  Nhits lengthRead strand           chr   pos
1     1         34      - CHR1v01212004 20729
2     1         34      - CHR1v01212004 62752
3     1         34      + CHR1v01212004  8248
4     1         34      + CHR1v01212004 48961
5     1         34      + CHR1v01212004  8248
6     1         34      - CHR1v01212004 55621

R> head(controlSEP3_test)

  Nhits lengthRead strand           chr   pos
1     1         35      - CHR1v01212004  5714
2     1         35      + CHR1v01212004 39819
3     1         35      - CHR1v01212004 34149
4     1         35      + CHR1v01212004 22988
5     1         35      - CHR1v01212004 83202
6     1         35      - CHR1v01212004 56583

R> nhitsS <- mappedReads2Nhits(sampleSEP3_test, file = "sampleSEP3_test",
     chr = c("CHR1v01212004"), chrL = c(10000))
R> nhitsC <- mappedReads2Nhits(controlSEP3_test,
     file = "controlSEP3_test", chr = c("CHR1v01212004"),
```

```
     chrL = c(10000))
R> nhitsC$filenames

[1] "CHR1v01212004_controlSEP3_test.CSARNhits"

R> nhitsS$filenames

[1] "CHR1v01212004_sampleSEP3_test.CSARNhits"
```

The variable nhitsC and nhitsS will have the needed information to use with the function *ChIPseqScore* in order to calculate the read-enrichment score of the sample compared to the control for each nucleotide position. The results are saved in one file per each chromosome. *sigWin* will generate candidate read-enriched regions, and *score2wig* will generate a wig file that can be read by standard genome browsers. *distance2Genes* function will report the relative position of candidate read-enriched regions regarding the start and end position of the annotated genes. *genesWithPeaks* function will report genes with a candidate enriched region located near them.

```
R> test <- ChIPseqScore(control = nhitsC, sample = nhitsS,
     file = "test", times = 10000)
R> test$filenames

[1] "CHR1v01212004_test.CSARScore"

R> win <- sigWin(test)
R> head(win)

            chr start  end posPeak score length
1 CHR1v01212004  2616 2624    2616  1.16      9
2 CHR1v01212004  8133 8575    8377  4.14    443

R> score2wig(test, file = "test.wig", times = 10000)
R> d <- distance2Genes(win = win, gff = TAIR8_genes_test)
R> head(d)

            peakName    p1    p2 score      gene   le
1 CHR1v01212004_2616 -1015 -3283  1.16 AT1G01010 2268
2 CHR1v01212004_8377   360 -1587  4.14 AT1G01020 1947

R> genes <- genesWithPeaks(d)
R> head(genes)

       name max3kb1kb u3000 u2000 u1000   d0 d1000
1 AT1G01010      1.16     0  1.16     0 0.00     0
2 AT1G01020      4.14     0  0.00     0 4.14     0
```

With each run of the function *permutatedWinScores* one set of permutated scores is generated. Later, we can get the distribution of score values with the function *getPermutatedWinScores*. From this distribution, several cut-off values can be calculated to control the error of our test using functions implemented in R. In this package, it is implemented a control of the error based on FDR using the function *getThreshold*.

```
R> permutatedWinScores(nn = 1, sample = sampleSEP3_test,
     control = controlSEP3_test, fileOutput = "test",
     chr = c("CHR1v01212004"), chrL = c(1e+05))
R> permutatedWinScores(nn = 2, sample = sampleSEP3_test,
     control = controlSEP3_test, fileOutput = "test",
     chr = c("CHR1v01212004"), chrL = c(1e+05))
R> nulldist <- getPermutatedWinScores(file = "test",
     nn = 1:2)
R> getThreshold(winscores = win$score, permutatedScores = nulldist,
     FDR = 0.05)

   threshold Error_type_I        FDR
20      2.98   0.02127660 0.04255319
```

This is a very simple function to obtain the threshold value of our test statistic controlling FDR at a desired level. Basically, for each possible threshold value, the proportion of error type I is calculated assuming that the permutated score distribution is a optimal estimation of the score distribution under the null hypothesis, and FDR is obtained as the ratio of the proportion of error type I by the proportion of significant tests. Other functions implemented in R (eg: *multtest*) could be less conservative.

References

[1] Kerstin Kaufmann, Jose˜M Muino, Ruy Jauregui, Chiara˜A Airoldi, Cezary Smaczniak, Pawel Krajewski, and Gerco˜C Angenent. Target genes of the mads transcription factor sepallata3: Integration of developmental and hormonal pathways in the *arabidopsis* flower. *PLoS Biol*, 7(4):e1000090, 04 2009.

[2] Kerstin Kaufmann, Jose˜M Muino, Magne Østerås, Laurent Farinelli, Pawel Krajewski, and Gerco˜C Angenent. Chromatin immunoprecipitation (chip) of plant transcription factors followed by sequencing (chip-seq) or hybridization to whole genome arrays (chip-chip). *Nature Protocols*, (accepted).

[3] Jose˜M Muino, Kerstin Kaufmann, Roeland˜C van Ham, Gerco˜C Angenent, and P˜Krajewski. Plant chip-seq analyzer: An r package for the statistical detection of protein-bound genomic regions. (submitted).

4 Details

This document was written using:

```
R> sessionInfo()

R version 2.12.1 (2010-12-16)
Platform: x86_64-pc-linux-gnu (64-bit)

locale:
 [1] LC_CTYPE=en_US.UTF-8       LC_NUMERIC=C
 [3] LC_TIME=en_US.UTF-8        LC_COLLATE=C
 [5] LC_MONETARY=C              LC_MESSAGES=en_US.UTF-8
 [7] LC_PAPER=en_US.UTF-8       LC_NAME=C
 [9] LC_ADDRESS=C               LC_TELEPHONE=C
[11] LC_MEASUREMENT=en_US.UTF-8 LC_IDENTIFICATION=C

attached base packages:
[1] stats     graphics  grDevices utils     datasets
[6] methods   base

other attached packages:
[1] CSAR_1.4.0

loaded via a namespace (and not attached):
[1] tools_2.12.1
```
